# Supplementary material for: The Impact of Telepresence Robots on Family Caregivers and Residents in Long-Term Care
Source: Int J Environ Res Public Health. 2025 May 1;22(5):713. doi: 10.3390/ijerph22050713 (PMC12110784; doi:10.3390/ijerph22050713)
Supplement: Supplementary file 1 [file ijerph-22-00713-s001.zip › Table S1 COREQ checklist.pdf]

Table S1. Consolidated Criteria for Reporting Qualitative Studies (COREQ): 32-item Checklist

**No. Item**	**Guide questions/description**	**Reported on Page #**
Domain 1: Research team and reflexivity		
Personal Characteristics		
1. Interviewer/facilitator	Which author/s conducted the interview or focus group?	Page 6
2. Credentials	What were the researcher's credentials? e.g., PhD, MD	Page 6
3. Occupation	What was their occupation at the time of the study?	Page 6
4. Gender	Was the researcher male or female?	Page 6
5. Experience and training	What experience or training did the researcher have?	Page 6
Relationship with participants		
6. Relationship established	Was a relationship established prior to study commencement?	Page 7 .
7. Participant knowledge of the interviewer	What did the participants know about the researcher? e.g., personal goals, reasons for doing the research	Page 7
8. Interviewer characteristics	What characteristics were reported about the inter viewer/facilitator? e.g., Bias, assumptions, reasons and interests in the research topic	Page 7
Domain 2: study design		
Theoretical framework		
9. Methodological orientation and Theory	What methodological orientation was stated to underpin the study? e.g., grounded theory, discourse analysis, ethnography, phenomenology, content analysis	Page 2, 3
Participant selection		
10. Sampling	How were participants selected? e.g., purposive, convenience, consecutive, snowball	Page 5
11. Method of approach	How were participants approached? e.g., face-to-face, telephone, mail, email	Page 5, 6
12. Sample size	How many participants were in the study?	Page 5

13. Non-participation	How many people refused to participate or dropped out? Reasons?	Page 5
Setting		
14. Setting of data collection	Where was the data collected? e.g., home, clinic, workplace	Page 5
15. Presence of non-participants	Was anyone else present besides the participants and researchers?	Page 5
16. Description of sample	What are the important characteristics of the sample? e.g., demographic data, date	Page 7, 8
Data collection		
17. Interview guide	Were questions, prompts, guides provided by the authors? Was it pilot tested?	page 3, 4
18. Repeat interviews	Were repeat interviews carried out? If yes, how many?	NA
19. Audio/visual recording	Did the research use audio or visual recording to collect the data?	Page 5
20. Field notes	Were field notes made during and/or after the interview or focus group?	Page 5
21. Duration	What was the duration of the interviews or focus group?	Page 5
22. Data saturation	Was data saturation discussed?	NA
23. Transcripts returned	Were transcripts returned to participants for comment and/or correction?	NA
Domain 3: analysis and findings		
Data analysis		
24. Number of data coders	How many data coders coded the data?	Page 6
25. Description of the coding tree	Did authors provide a description of the coding tree?	Page 11
26. Derivation of themes	Were themes identified in advance or derived from the data?	Page 11-13
27. Software	What software, if applicable, was used to manage the data?	Page 6
28. Participant checking	Did participants provide feedback on the findings?	NA
Reporting		
29. Quotations presented	Were participant quotations presented to illustrate the themes/findings? Was each quotation identified? e.g., participant number	Page 11-13

30. Data and findings consistent	Was there consistency between the data presented and the findings?	Page 11-13
31. Clarity of major themes	Were major themes clearly presented in the findings?	Page 11-13
32. Clarity of minor themes	Is there a description of diverse cases or discussion of minor themes?	Page 11-13

Note. NA = not applicable.

Developed from:
Tong, A.; Sainsbury, P.; & Craig, J. Consolidated criteria for reporting qualitative research (COREQ): A 32-item checklist for interviews and focus groups. *International Journal for Quality in Health Care* **2007**, *19(6)*, 349-357, doi:10.1093/intqhc/mzm042
