# Supplementary material for: The Impact of Telepresence Robots on Family Caregivers and Residents in Long-Term Care
Source: Int J Environ Res Public Health. 2025 May 1;22(5):713. doi: 10.3390/ijerph22050713 (PMC12110784; doi:10.3390/ijerph22050713)
Supplement: Supplementary file 1 [file ijerph-22-00713-s001.zip › Table S2 GRAMMS checklist.pdf]

Table S2. Good Reporting of A Mixed Methods Study (GRAMMS) checklist

**Guideline**	**Reported on page #**
Describe the justification for using a mixed methods approach to the research question	Page 2-3
Describe the design in terms of the purpose, priority and sequence of methods	Page 2-3
Describe each method in terms of sampling, data collection and analysis	Page 3-7
Describe where integration has occurred, how it has occurred and who has participated in it	Page 7
Describe any limitation of one method associated with the present of the other method	Page 14-16
Describe any insights gained from mixing or integrating methods	Page 14-16

Developed from:

O'Cathain, A., Murphy, E., & Nicholl, J. (2008). The quality of mixed methods studies in health services research. Journal of Health Services Research & Policy, 13(2), 92-98. https://doi.org/10.1258/jhsrp.2007.007074
